# A preliminary examination of the relationship between specific adverse childhood experiences and perpetration of hate-motivated behaviours

**DOI:** 10.1177/00207640251353819

**Published:** 2025-07-26

**Authors:** Sofia Desogus, Kirsten Russell

**Affiliations:** Department of Psychological Sciences and Health, University of Strathclyde, Glasgow, UK

**Keywords:** Hate motivated behaviour, hate crime, microaggressions, adverse childhood experiences

## Abstract

**Background:**

Hate-motivated behaviour (HMB) is a growing public health issue. These behaviours can range from hate crimes to microaggressions and have been associated with wide-ranging consequences.

**Aims:**

The current study aimed to explore the relationship between specific adverse childhood experiences (ACEs) and HMB perpetration.

**Methods:**

Participants (*n* = 447) completed an online cross-sectional survey assessing demographic factors, ACEs and HMB perpetration.

**Results:**

Individuals who experienced ACEs were more likely to report engaging in HMB than those who did not experience negative life events during childhood. Abuse, neglect and living with a family member with substance abuse and/or mental health issues were all associated with HMB perpetration.

**Conclusions:**

This study provides a more nuanced understanding of the link between experiences during childhood and HMB later in life, by highlighting that specific ACEs were more strongly associated with engaging in these behaviours. Future research should seek to replicate these findings and examine the mechanisms underpinning these relationships.

## Background

Hate Motivated Behaviour (HMB) is a growing public health issue (Scottish Government, 2023) and includes subtypes, ranging from hate crimes to behaviour that falls short of illegality, such as microaggressions (Cramer et al., 2021). They have been associated with poor psychological, social and emotional health outcomes (Chakraborti et al., 2014) and at a societal level, can be socially divisive, and can heighten tensions between communities (Hall, 2013). The current study aims to expand on existing research regarding factors that may explain why some people engage in HMB and others do not by examining the relationship between Adverse Childhood Experiences (ACEs) and perpetration of HMB.

ACEs, such as abuse, neglect or living with a parent who engages in harmful drug use, are recognised as events that pose a long-term threat to mental and physical health (Felitti et al, 1998) and research has demonstrated that these experiences are associated with adverse social and behavioural outcomes, including engagement in violent behaviours (Levenson, 2014; Perfect et al., 2016). Whilst existing literature on ACEs has largely focused on those who have engaged in *criminal* activity (Lensch et al., 2021), common HMBs are not considered to be criminal offences (Equality and Human Rights Commission, 2011). As such, community-based research examining the link between ACEs and HMB is required.

Most research categorises individuals, into high-risk and low risk for experiencing negative consequences, based on the number of ACEs they have experienced. However, this approach does not allow for differentiation of experience, and does not account for the fact that different ACEs may have different impacts on those who experience them (i.e. some ACEs may be more likely to be associated with poor outcomes than others). A more nuanced understanding of this link has the potential to inform risk assessment as well as HMB prevention efforts. As such, the current study aimed to investigate which specific ACEs are associated with HMB perpetration within a community-based sample.

## Methodology

### Procedure

Given the preliminary nature of this research a cross-sectional study was implemented. The sample was drawn from a nationwide survey study investigating experiences of HMB. This investigation adhered to the British Psychological Society’s ethical guidelines and approval was obtained from the University Ethics Committee (#UEC21/83). Participants were provided with an information sheet. Informed consent was requested before participants were able to access the survey. Data was collected online via self-report questionnaires. Participants were asked to complete a basic demographic questionnaire followed by two measures, presented in a randomised order. A downloadable debrief sheet was provided upon completion of the survey.

### Participants

Table 1 contains sample demographic information. The sample was predominantly comprised of White Scottish (79.4%), female (79.0%), young adults (average age = 23 years old).

**Table 1.** Sample demographic information.

Variable	*n* (%)	*M* (*SD*)
Age	–	22.84 (7.27)
Estimated annual income	–	£13,346.98 (£12,620.58)
Gender		
Male	76 (17.0)	–
Female	353 (79.0)	–
Non-binary	9 (2.0)	–
Transgender female	4 (0.9)	–
Transgender male	2 (0.4)	–
Queer	3 (0.7)	–
National identity		
Scottish	372 (83.2)	–
English	33 (7.4)	–
Northern Irish	8 (1.8)	–
British	3 (0.7)	–
Malaysian	5 (1.1)	–
Irish	4 (0.9)	–
Omani	3 (0.7)	–
Other	18 (4.0)	–
Missing	1 (0.2)	–
Ethnicity/race		
White Scottish	355 (79.4)	–
White Irish	8 (1.8)	–
White other British	29 (6.5)	–
White Polish	2 (0.4)	–
Asian/Asian Scottish/Asian British	21 (4.7)	–
African/African Scottish/African British	2 (0.4)	–
Biracial	3 (0.7)	–
Multiracial	7 (1.6)	–
Other White (e.g. Austrian)	12 (2.7)	–
Other	6 (1.3)	–
Missing	2 (0.4)	–
Sexual orientation		
Gay	10 (2.2)	–
Lesbian	16 (3.6)	–
Heterosexual	300 (67.1)	–
Bisexual	64 (14.3)	–
Queer	15 (3.4)	–
Questioning	6 (1.3)	–
Pansexual	13 (2.9)	–
Asexual	3 (0.7)	–
Prefer no label	19 (4.3)	–
Missing	1 (0.2)	-–
Political affiliation		
Scottish national party	194 (43.4)	–
Conservative	10 (2.2)	–
Labour	96 (21.5)	–
Liberal democrats	16 (3.6)	–
Green party	57 (12.8)	–
Other (e.g. none)	56 (12.5)	–
Missing	18 (4.0)	–
Religion		
Catholic	98 (21.9)	–
Protestant	49 (11.0)	–
Baptist	1 (0.2)	–
Christian other	20 (4.5)	–
Muslim	10 (2.2)	–
Buddhist	3 (0.7)	–
Atheist	124 (27.7)	–
Agnostic	61 (13.6)	–
Spiritual	24 (5.4)	–
Other (e.g. no religion)	17 (3.6)	–
Prefer not to say	33 (7.4)	–
Missing	7 (1.6)	–

*Note.* *N* = 447; *M* = Mean; *SD* = Standard deviation.

### Measures

#### Hate-motivated behaviour

History of engaging in HMB was captured using the Hate-Motivated Behaviour Checklist (HMBC; Cramer et al., 2021; α = .88). Participants were asked how often they had engaged in 26 specific HMB that encompass violence, property crime and noncriminal micro-aggressive acts. The HMBC was chosen as it provides the opportunity to assess the full range of HMB (from non-criminal to criminal) and from a perpetrator perspective.

#### Adverse childhood experience

The 10-item version of the Adverse Childhood Experiences Questionnaire was implemented to capture participants exposure to negative life experiences during the first 18 years of life (α = .88). This measure was chosen as it has been widely used in previous research and, therefore, provides the opportunity for this work to contribute to a cumulative evidence base on the consequences of experiencing ACEs (Bellis et al., 2019).

### Analytical plan

To determine whether individuals who had experienced ACEs were more likely to have perpetrated HMB, a multiple linear regression was performed (controlling for age and gender). Further exploratory analyses, using separate linear regressions, were conducted to examine whether specific ACEs were more likely to be associated with HMB perpetration than others.^1^

## Results

Age, gender and total number of ACES explained 37% of the variance (*R*^2^ = .37, *F* (3, 368) = 19.34). Individuals who experienced ACEs were significantly more likely to report HMB perpetration compared to those that had not experience these negative life events (β = .27, *p* < .001).

Emotional abuse (β = .23, *p* < .001), physical abuse (β = .25, *p* < .001), sexual abuse (β = .14, *p* < .001), emotional neglect (β = .19, *p* < .001), physical neglect (β = .16, *p* < .001), family substance abuse (β = .20, *p* < .001) and living with a family member with mental health issues (β = .25, *p* < .001) significantly associated with HMB perpetration. However, parental divorce or separation, witnessing domestic violence and having a household member who was incarcerated were not (Table 2).

**Table 2.** Results of linear regression analyses examining the association between ACEs categories and HMB perpetration.

ACE categories	*B*	*SE B*	β	*t*	*p*
Emotional abuse	2.07	0.411	.233	5.05	<.001
Physical abuse	2.71	0.487	.255	5.57	<.001
Sexual abuse	2.36	0.768	.145	3.08	.002
Emotional neglect	1.71	0.424	.189	4.05	<.001
Physical neglect	2.43	0.723	.158	3.37	<0.001
Parental divorce/separation	0.364	0.395	.044	0.920	.358
Witnessing domestic violence	1.48	0.612	.114	2.42	.016
Family substance abuse issues	1.86	0.442	.197	4.22	<.001
Family mental health issues	1.21	0.386	.147	3.13	.002
Incarcerated family member	2.08	0.892	.110	2.34	.020

## Discussion

The study aimed to investigate the relationship between ACEs and the perpetration of HMB within a community-based sample in Scotland. We sought to extend the existing literature by exploring which specific ACEs were associated with increased risk of engaging in these hateful acts.

Within our sample, 69% of perpetrators of HMB had experienced at least one ACE. Furthermore, as the number of ACEs experienced by participants increased, so too did the likelihood that they would report having engaged in HMB in their lifetime. Our results are in accordance with previous research that has suggested that individuals engage in criminal or violent activity are more likely to have experienced negative life events during childhood (Baglivio et al., 2014). These findings extend the literature by highlighting, for the first time, that ACEs are associated with HMB (measured on a continuum ranging from microaggressions to hate crimes).

Past studies have examined associations between specific ACE categories and health outcomes later in life (e.g. Chang et al., 2019). Exploratory analysis was conducted to evaluate the differential impact of individual ACE categories on the risk of perpetrating HMB later in life and in doing so extends the existing literature. It does so by, providing a more in-depth analysis of which early experiences are more likely to predict HMB. All ACEs, except for parental divorce or separation, witnessing domestic violence and having an incarcerated household member, were linked to HMB perpetration. It has been proposed that childhood trauma can affect multiple interconnected neurobiological systems, and that this can have consequences for emotional and behavioural regulation (Katembu et al., 2023). In turn this may mean that these individuals are more likely to become involved in violence and criminal activity (including HMB).

The current research set out to examine the relationship between HMB and ACEs. ACEs, both individually and cumulatively, were found to be associated with an increased likelihood of perpetrating HMB. The exploratory analysis extends the extant literature, allowing for a deeper and more nuanced understanding of which early experiences are more likely to predict HMB. Replication of these findings is warranted as identifying ACEs as predictors of HMB is has potential implications for risk assessment and intervention strategies that can be applied with those who have experienced ACEs. With regards to risk assessment there may be the potential for early identification of individuals at risk of engaging in HMB based on their ACE profiles. For those who have experienced ACEs, interventions to improve parenting skills, to strengthen parent-child attachment and to develop children’s resilience can help to moderate the harmful effects of ACEs (Bellis et al., 2019).

Study limitations include a cross-sectional, online self-report survey design and procedure, as well as a restricted sample with respect to age and race. Future HMB research may employ community-engaged and multi-method approaches to obtain more diverse samples in Scotland. Future research should also distinguish between different types of HMB, as the psychological mechanisms linking ACEs to microaggressions may differ from those leading to violent hate crimes. Moreover, an investigation into race-based ACEs and their influence on HMB would be particularly valuable in understanding how early experiences of racism shape later behaviours.

## Notes

1. A Bonferroni correction was applied to adjust for conducting multiple tests.
